# Mn(II)-Tagged DOTA-Modified Sugar-Based Biopolymers as Gadolinium-Free Contrast Agents for Magnetic Resonance Imaging

**DOI:** 10.3390/pharmaceutics18050530

**Published:** 2026-04-27

**Authors:** Irena Pashkunova-Martic, Joachim Friske, Silvester J. Bartsch, Daniela Prinz, Theresa Balber, Verena Pichler, Dieter Baurecht, Bernhard K. Keppler, Thomas H. Helbich

**Affiliations:** 1 Department of Biomedical Imaging and Image-Guided Therapy, Division of Molecular and Structural Preclinical Imaging, Medical University of Vienna & General Hospital of Vienna, Waehringer Guertel 18-20, 1090 Vienna, Austria; joachim.friske@meduniwien.ac.at (J.F.); silvester.bartsch@tum.de (S.J.B.); daniela.a.prinz@meduniwien.ac.at (D.P.); thomas.helbich@meduniwien.ac.at (T.H.H.); 2 Institute of Inorganic Chemistry, Faculty of Chemistry, University of Vienna, Waehringer Strasse 42, 1090 Vienna, Austria; bernhard.keppler@univie.ac.at; 3 Department of Nuclear Medicine, TUM Klinikum Rechts Der Isar, Technical University of Munich, 80333 Munich, Germany; 4 Department of Biomedical Imaging and Image-Guided Therapy, Division of Nuclear Medicine, Medical University of Vienna & General Hospital of Vienna, Waehringer Guertel 18–20, 1090 Vienna, Austria; theresa.balber@meduniwien.ac.at; 5 Joint Applied Medicinal Radiochemistry Facility, University of Vienna and Medical University of Vienna, 1090 Vienna, Austria; verena.pichler@univie.ac.at; 6 Department of Pharmaceutical Sciences, University of Vienna, Josef-Holaubek-Platz 2 (UZA II), 1090 Vienna, Austria; 7 Department of Pharmacy, University of Oslo, Problemveien 11, 0313 Oslo, Norway; 8 Department of Physical Chemistry, Faculty of Chemistry, University of Vienna, Waehringer Strasse 42, 1090 Vienna, Austria; dieter.baurecht@univie.ac.at

**Keywords:** chitosan oligosaccharide, dextran, manganese, gadolinium-free contrast agents, MRI

## Abstract

**Background**: Paramagnetic manganese (Mn(II)) has emerged as a promising alternative to gadolinium-based contrast agents (GBCAs) due to its favorable magnetic properties. Despite extensive research, no Mn-based agent has yet achieved clinical translation. Because free Mn(II) is toxic, macromolecular complexes incorporating stable macrocyclic DOTA chelators conjugated to polysaccharides may enhance coordination stability and improve the safety profile of Mn(II)-based contrast agents. **Methods**: Two chemical routes, maleimide- and ester-mediated, were evaluated for covalent coupling of DOTA-based macrocyclic ligands to the backbone of selected poly- and oligosaccharides. Subsequently, DOTA-modified carboxymethyldextran, aminodextran, and chitosan oligosaccharide were labeled with paramagnetic Mn(II) under mild conditions. ATR-FTIR confirmed the successful conjugation of DOTA chelators to the sugar backbone. The conjugates were further characterized by DLS, ICP-MS, and FPLC. In vitro relaxivity was measured at high field strength to evaluate MRI performance. In vivo contrast efficacy was first assessed using in ovo MRI in chicken embryos and subsequently evaluated by biodistribution studies in nude mice. **Results**: In vitro relaxivity studies demonstrated higher signal enhancement of the poly-/oligosaccharide-DOTA-Mn(II) conjugates compared with MnCl_2_ and the clinical agent gadoteridol (ProHance^®^). In ovo MRI showed persistent vascular enhancement up to 120 min, while in nude mice, contrast enhancement was observed in the liver, kidneys, and gallbladder 40 min post-injection. **Conclusions**: Mn(II)-tagged sugar-based imaging probes may offer a promising non-gadolinium alternative to GBCAs, with tunable biodistribution profiles depending on carrier molecular weight.

## 1. Introduction

MRI is a powerful, non-invasive diagnostic tool that enables very high spatial and temporal resolution and can provide detailed molecular information when combined with a contrast agent with high relaxivity to overcome the lack of sensitivity inherent to MRI [1]. However, millions of doses of applied GBCAs result in an enormous environmental burden. GBCAs have been detected in surface and drinking water in recent years [2]. The free Gd(III) ion has been identified as an aquatic microcontaminant that poses a great threat to water organisms and human beings and thus requires serious attention. Unintentional human intake of Gd(III) leads to detrimental health issues such as digestive symptoms, twitching or weakness, cognitive fog, persistent skin diseases, body pains, acute renal and non-renal adverse reactions, and chronic skin and eye changes [2,3]. Furthermore, accumulation of bound and free Gd(III) in different organs of the human body has been observed and is problematic because of potential health concerns and even potentially fatal diseases, including nephrogenic systemic fibrosis (NSF) [4]. Recent studies have reported on Gd(III) deposition in neural tissue, and in muscles and bones in patients with normal kidney function who have undergone serial contrast-enhanced MRI examinations [5,6,7]. Moreover, the only approved and clinically used GBCAs are contraindicated in patients with acute and chronic renal impairment. Thus, there is a critical and unmet clinical need for safer forms of MRI contrast agents applicable in the setting of renal insufficiency or other contraindications. Thus, research on Gd(III) alternatives is currently gaining significant attention and the development of new Gd-free MRI contrast agents (CA) with safer profiles is a pressing research goal in diagnostic imaging.

In particular, Mn(II) and its paramagnetic complexes are an attractive alternative class of MRI contrast agents. Mn(II) is an important nutrient for proper health and maintenance in the human body and is an essential trace element in human physiology, which has many favorable properties that lead to its potential use as an MRI CA: high spin number; long electronic relaxation time; and labile water exchange [8]. Although manganese is a trace element in the human body for which all the regulation mechanisms are already present, free Mn(II) ions in high concentrations may exert serious side effects. Therefore, it is important to bind Mn(II) ions to ligands that not only prevent interactions between the metal cations and endogenous tissue (including blood components) but also decrease the T1 shortening effect by limiting the proximity of Mn(II) ions to protons [9].

Aware that DOTA-like ligands occupy all coordination sites of Mn(II), leaving no possibility for the attachment of inner-sphere water molecules, we aimed to design a stable cage-like complex with the known high kinetic inertness of [Mn(II)-DOTA]^2−^ [10] and to enhance the longitudinal relaxivity (r_1_) by slowing down the rotational dynamics of the entire molecule. To achieve this, we considered covalent coupling to macromolecules such as DOTA-modified sugar-based polymers. Such macromolecular conjugation reduces the molecular tumbling rate of the overall construct, resulting in increased relaxivity.

One potential macromolecular scavenger and carrier for paramagnetic manganese complexes explored in this study is dextran (carboxymethyldextran, CMD, and the lysine-functionalized or aminodextran, AD). Dextrans are a family of natural polysaccharides containing negatively charged groups and have been widely investigated as polymeric carriers in novel drug delivery systems [11]. Numerous efforts have been directed toward the development of dextran conjugates and their biomedical applications. However, the major drawback of dextran, which limits its utility as a drug delivery platform, is its susceptibility to enzymatic digestion in the human body and its high polarity, which prevents transcellular passage [12]. This limitation can be overcome by using thiolated polysaccharides or polymers (thiomers), or hydrophilic polymers modified with thiol moieties, which exhibit high transfection efficiency and mucoadhesive properties [13].

Chitosan, an oligosaccharide, is another natural sugar-based biopolymer with lower molecular weight, high biodegradability, and excellent biocompatibility, offering attractive physicochemical properties for binding paramagnetic ions [14,15]. Nevertheless, its limited solubility and low buffering capacity at physiological pH lead to reduced transfection efficiency. The presence of amine functional groups enables interactions with negatively charged macromolecules or organic ligands, allowing the formation of biodegradable and tunable scaffolds for diverse biomedical applications, including nucleic acid and drug delivery systems [16]. These characteristics make chitosan a highly promising material for biomedical research.

In this study, three biopolymer-based macromolecular Mn(II) complexes: CMD–DOTA–Mn(II), AD–DOTA–Mn(II), and CH–DOTA–Mn(II) were synthesized at high yields through reproducible covalent coupling strategies. Characterization by ATR–FTIR confirmed successful ligand functionalization, while DLS analysis demonstrated well-defined hydrodynamic sizes consistent with stable macromolecular conjugates. ICP–MS quantification indicated efficient Mn loading in all formulations. The use of different sugar backbones allowed modulation of molecular weight and structural properties, providing a useful platform for investigating structure–function relationships in macromolecular MRI agents.

## 2. Materials and Methods

### 2.1. Chemicals

Carboxymethyl dextran (70 kDa) was provided by TdB Labs, Uppsala, Sweden, whereas MnCl_2_ × 4H_2_O, lysinated aminodextran (70 kDa), L-cysteine, HEPES (4-(2-hydroxyethyl)piperazine-1-ethanesulfonic acid), and EDAC (1-Ethyl-3-(3-Dimethylaminopropyl) carbodiimide, Hydrochloride) were purchased from Sigma-Aldrich, Vienna, Austria. Maleimide-DOTA-GA and p-NCS-Bz-DOTA-GA were supplied by ChemaTech, Dijon, France. Oligo-chitosan (5 kDa), highly deacetylated for medical purposes, was purchased from Heppe Medical Chitosan, HPC^+^, Halle, Germany. Ultrapure water (18.2 MΩ cm, ELGA Water purification system, Purelab Ultra MK 2, UK, or 18.2 MΩ cm, MilliQ Advantage, Darmstadt, Germany), HNO_3_ (≥69%, Rotipuran Supra, Carl Roth, Karlsruhe, Germany), and H_2_O_2_ (30%, Suprapur, Merck, Darmstadt, Germany) were used for all ICP-MS measurements.

### 2.2. Methods

#### 2.2.1. Preparation of CMD-DOTA-Mn(II)

##### Preparation of Thiolated CMD and Direct Mn(II)-Labeling of CMD-SH via Maleimide-DOTA-GA

CMD (100 mg, MW 70 kDa; 0.05 mM) was dissolved in Milli-Q^®^ water (30 mL) in triplicate. EDAC (345 mg) was added to each dextran solution, and the reaction mixtures were stirred for 45 min at room temperature (RT). An equimolar amount of L-Cys·HCl was then added, and the pH was adjusted to 4.0 using 1 M HCl. The mixtures were stirred for an additional three hours at RT. The resulting CMD-SH solutions were purified by FPLC using two Sephadex G25 columns (Cytiva^®^, Freiburg, Germany; HiTrap Desalting), and the collected fractions were lyophilized to yield CMD-SH.

**Scheme 1:**
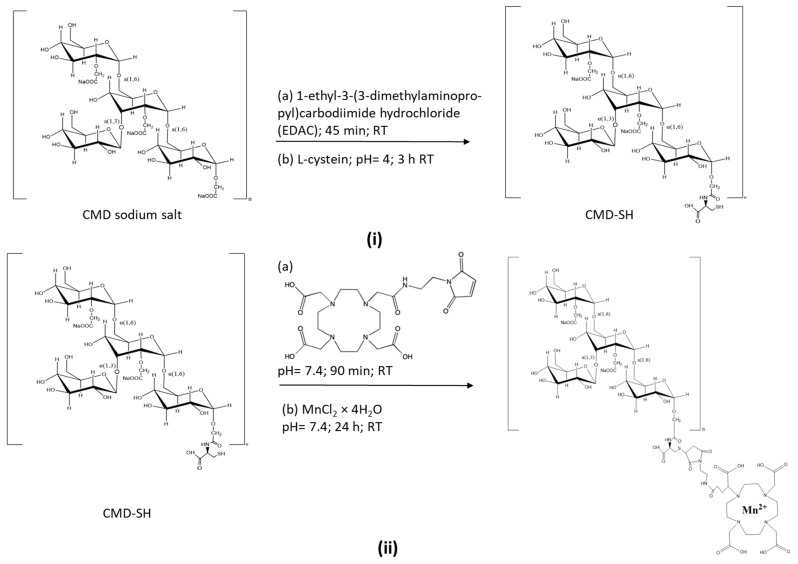
Synthetic scheme of the preparation of CMD-SH by EDAC-mediated conjugation and coupling of L-cysteine (**i**), followed by complexation with Mn(II) via maleimide-DOTA-GA (**ii**).

Aliquots of CMD-SH (10 mg) were subsequently reacted with a fivefold molar excess of maleimide-DOTA-GA. The mixtures were stirred for 90 min at RT to afford CMD-DOTA-maleimide (Scheme 1). The final conjugate was purified and isolated by FPLC as described in Section 2.2.3 in detail. Mn(II) complexation was performed by adding MnCl_2_·4H_2_O solution (0.05 mL, 0.2 M) to each DOTA-modified oligo- or polysaccharide solution. The reaction mixtures were stirred for 24 h at RT to ensure complete complexation. The end product was further purified by FPLC (see Section 2.2.3).

#### 2.2.2. Preparation of AD-DOTA-Mn(II) and CH-DOTA-Mn(II) via p-NCS-Bz-DOTA-GA

Poly- and oligosaccharides (30 mg each; MW 70 and 5 kDa, respectively) were dissolved in HEPES buffer (0.5 mL, 100 mM, pH 8.4) in triplicate. A 20-fold molar excess of p-NCS-Bz-DOTA was suspended in DMSO (0.04 mL) and added stepwise to the sugar solutions. After each addition, the pH was adjusted to 8.5 using 0.3 M NaOH. Following the final addition, the reaction mixtures were stirred for one hour at RT.

**Scheme 2:**
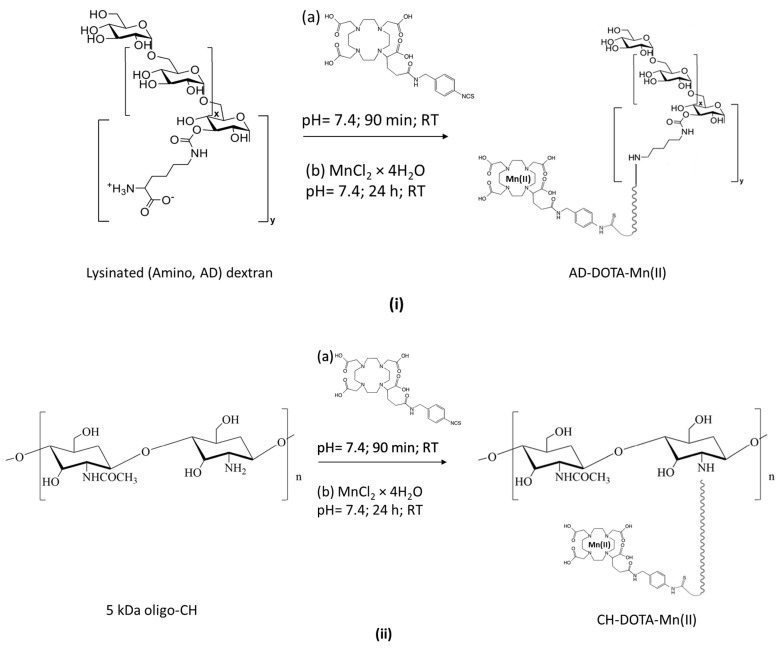
Synthetic schemes of the preparation of AD-DOTA-Mn(II) (**i**) and CH-DOTA-Mn(II) (**ii**) via conjugation with p-NCS-Bz-DOTA and their subsequent complexation with Mn(II).

To remove unbound DOTA ligand, the reaction mixtures were purified by FPLC. In detail, high-molecular-weight CMD- and AD-based conjugates were purified using two Sephadex G25 columns (Cytiva^®^, Freiburg, Germany; HiTrap Desalting; column volume 5 mL each; exclusion limit Mr 5000) connected in series. The CH-DOTA intermediate was purified using a Superdex 75 HR 10/30 column (Cytiva^®^, Freiburg, Germany; HiTrap Desalting; column volume 24 mL; separation range Mr 3000–70,000). The applied sample volume was 0.5 mL, and the flow rate was 0.5 mL min^−1^. Citrate buffer (100 mM, pH 6.5) was used as the eluent for all three oligo/polysaccharidic carriers. All chromatographic separations were performed using an ÄKTA™ purifier 10 system (Cytiva^®^). Fractions containing poly-/oligosaccharide–DOTA conjugates were collected (2 mL for CMD- and AD-dextran conjugates, 8 mL for CH-DOTA) and subsequently used for complexation with Mn(II) subsequently used for complexation with Mn(II) (Scheme 2).

#### 2.2.3. Isolation and Purification by Fast Protein Liquid Chromatography (FPLC)

Unreacted EDAC and L-cysteine were removed, and the CMD-SH conjugate was isolated by FPLC using an ÄKTA™ purifier 10 system (Cytiva^®^), as described above. Polysaccharide fractions containing CMD-SH (2–3 mL each) were collected and lyophilized until further analysis or derivatization.

Prior to mass spectrometric analysis, the final Mn(II) conjugates (0.010 g each, dissolved in 0.5 mL of distilled water) were further purified by size exclusion chromatography using two Sephadex G-25 superfine columns (Cytiva^®^; 5 mL each) connected in series. The applied sample volume was 0.5 mL, and the flow rate was maintained at 1.0 mL min^−1^. The isolated fractions were lyophilized, and the Mn content was determined by ICP–MS (see Section 2.2.5).

#### 2.2.4. Attenuated Total Reflectance-Fourier Transform Infrared Spectroscopy (ATR-FTIR)

ATR–FTIR spectra of the starting CMD and AD, CMD-SH, CMD-SH-DOTA, AD-maleimide-DOTA intermediates, and the final CMD-SH-DOTA-Mn(II) and AD-p-NCS-DOTA-Mn(II) conjugates were recorded on a Bruker Tensor 37 FTIR spectrometer equipped with a Bruker Platinum ATR accessory and a single-reflection diamond crystal. Spectra were collected over the range of 4000–400 cm^−1^ at a resolution of 4 cm^−1^ and averaged over 128 scans. Lyophilized CMD was analyzed as received from the manufacturer. All spectra were recorded in the lyophilized state, using a clean diamond surface as the reference background.

#### 2.2.5. Dynamic Light Scattering (DLS)

The hydrodynamic diameter and surface potential (zeta potential, **ζ**) of the Mn(II) sugar-based conjugates were determined by dynamic light scattering (DLS), using a Zetasizer Nano series instrument instrument (Nano-ZS, Malvern Panalytical, Malvern, UK). Measurements were performed in nanopure water as the dispersant (viscosity = 0.8872 cP; refractive index = 1.330) at a constant temperature of 25 °C. Sample concentrations ranged from 2 to 4 mg mL^−1^, yielding an average measurement intensity of approximately 330 Hz. Each sample was measured for a total of 160 min over three cycles, with a run time of four minutes min per cycle, and data were collected until convergence was achieved. The reported values represent the mean of three independent samples, each measured in triplicate. Zeta potential measurements were performed on the same instrument following calibration with a Zeta Potential Transfer Standard (−40 mV ± 5.8 mV).

#### 2.2.6. Determination of Mn(II) Content and Biodistribution in Mice Organs by Inductively Coupled Plasma-Mass Spectrometry (ICP-MS)

Standard solutions were purchased from Labkings (Hilversum, The Netherlands). The measurements were carried out on an Agilent 8800 ICP-MS/MS (Agilent Technologies, Tokyo, Japan), equipped with a CETAC ASX-520 autosampler (CETAC Technologies, Omaha, NE, USA) and a MicroMist nebulizer at a sample uptake rate of approx. 0.25 mL min^−1^. The Agilent MassHunter software package (Workstation Software, Version C.01.03, 2016) was used for data evaluation.

Determination of Mn(II) content: Aliquots (0.1 mL) of the purified poly- or oligosaccharide solutions were weighed, appropriately diluted, and brought to a final volume of 10 mL with 3% HNO_3_ prepared in Milli-Q^®^ water. The Mn content of the samples was determined using an ICP–MS/MS instrument (Agilent 8800; Agilent Technologies, Tokyo, Japan) equipped with a MicroMist nebulizer, operating at a sample uptake rate of approximately 0.25 mL min^−1^.

Biodistribution of Mn(II) in mouse organs: Prior to ICP–MS analysis, excised organs (from sixteen mice) were weighed (105–528 mg) for tissue digestion. Individual kidneys were placed in separate PFA tubes, while livers were divided into three to four fractions and transferred to the PFA tubes. To each tube, 4 mL of 20% HNO_3_ and 200 µL of H_2_O_2_ were added. The suspensions were placed on a hot plate and digested for six hours using a temperature program with a maximum temperature of 200 °C. After cooling, the clear solutions were transferred to 15 mL tubes. Residual liquid in the PFA tubes was recovered by washing with 4 mL of ultrapure water and combined with the corresponding digests.

All samples were subsequently diluted 1:3 with Milli-Q^®^ water to yield a final volume of approximately 10 mL and a final HNO_3_ concentration of 3% (*v*/*v*) prior to ICP–MS measurement. The dilution factor was calculated based on the total weight of each resulting sample solution.

#### 2.2.7. In Vitro MRI—Determination of Contrast Agent Relaxivity (r_1_)

To determine the longitudinal relaxivity (r_1_), serial dilutions of the poly-/oligosaccharide–DOTA–Mn(II) conjugates were prepared in PBS (pH 7.4). Initial concentrations were 0.2 × 10^−3^ M for AD–DOTA–Mn(II), 0.3 × 10^−3^ M for CMD–DOTA–Mn(II), and 0.2 × 10^−3^ M for CH–DOTA–Mn(II). A series of dilutions was prepared to identify the optimal concentration range for MRI measurements. Pure PBS was used as a reference for signal-to-noise ratio (SNR) calculations, while MnCl_2_ and gadoteridol (ProHance^®^, Milano, Italy) served as positive controls.

Longitudinal relaxation times (T_1_) were measured using a spin echo inversion and saturation recovery sequence. For each agent, four dilutions were prepared in PBS (1.5 mL per sample). Samples were placed in standard PE tubes (diameter 0.8 cm). MRI measurements were performed on a 9.4 T system using a transmit/receive volume coil (diameter 86 mm) and a saturation recovery sequence (T_1_-map RAREVTR). Variable acquisition times (TR = 6000, 7000, 100, and 50 ms) were used to generate the saturation recovery series. Other acquisition parameters included TE = 7 ms, RARE factor = 2, 10 averages, slice thickness = 1 mm, and in-plane resolution = 0.200 mm^2^. T_1_ maps were calculated using Bruker Paravision v360.336 and the build function in ISA Tool.

For quantitative SNR analysis and T_1_ evaluation, regions of interest (ROIs) were defined within each PBS-filled tube and in the background (noise). Mean signal intensities and corresponding standard deviations (SD) were extracted from ROI-based analysis of T_1_ maps generated in Paravision, with ROIs evaluated across multiple slices of the same sample. Relaxation rates were calculated as R_1_ = 1/T_1_, and uncertainties were propagated using standard Gaussian error propagation.

#### 2.2.8. Short-Term Stability Studies

Short-term stability of CMD–DOTA–Mn(II) and CH–DOTA–Mn(II) was evaluated in physiological saline (0.9% NaCl) over seven days at 4 °C (in the dark) and at room temperature (RT). Visual inspection was performed to assess potential turbidity or precipitation.

Hydrodynamic size distributions were monitored by dynamic light scattering (DLS) at day 0 and after seven days. Relaxometric stability was assessed by measuring longitudinal relaxation rates (R_1_) and corresponding relaxivities (r_1_) under identical conditions using the same MRI protocol as described in Section 2.2.6.

#### 2.2.9. In Ovo MRI

To evaluate in vivo distribution and radiological relevance, in ovo MRI prescreening was performed using fertilized chicken eggs on embryonal development day 17. For this purpose, fertilized hen’s eggs (n = 9) were incubated in a horizontal position using a dedicated breeding machine (60–65% relative humidity, 37 °C). Eggs were windowed on embryonal development day (EDD) 3 and windows were continuously enlarged until the day of imaging to provide access to the chorioallantoic membrane (CAM). Chicken embryos were anesthetized using 2–3% isoflurane in air to avoid motion artifacts during image acquisition. MR imaging was performed before and after injection of prepared poly-/oligosaccharide-based contrast agents. AD-DOTA-Mn(II), CMD-DOTA-Mn(II), and CH–DOTA–Mn(II) (0.07 mM Mn, 0.9 mM, and 0.4 mM Mn, respectively) were injected (three eggs per compound, injection volumes 100 µL) into a CAM-vessel using a self-made catheter, pre-flushed with physiological saline as previously described [17].

#### 2.2.10. Cell Viability Test

The CH–DOTA–Mn(II) formulation was selected for testing on healthy fibroblast cells because its relatively lower molecular mass, compared to the other two dextran-based formulations, was expected to facilitate rapid renal excretion in vivo.

*Cell line and cell culture conditions*

The immortalized adherent NIH-3T3 cells (clonal mouse fibroblast cell line) derived from randomly bred mice (CLS cell lines, Eppelheim, Germany) were grown in Dulbecco’s Modified Eagle’s Medium (DMEM)—high glucose and supplemented with 1% penicillin-streptomycin mixture (P/S), 1% L-glutamine (L-Glu) and 10% fetal calf serum (FCS). Cells were grown as an adherent monolayer culture at 37 °C in a humidified atmosphere and 5% CO_2_.

*Cell viability assay*

Cytotoxicity was determined by the colorimetric MTT assay (3-(4,5-dimethyl-2-thiazolyl)-2,5-diphenyl-2H-tetrazoliumbromide). 4 × 10^3^ NIH-3T3 cells per well and 100 µL were seeded in 96-well plates and cells were allowed to settle for 24 h. Stock solutions of CH-DOTA-Mn(II) were prepared in fully supplemented cell culture media with 1% DMSO. Cells were treated with 100 µL of a dilution series of CH-DOTA-Mn(II) with the concentrations 1, 0.5, 0.25, 0.125, 0.063, 0.031, 0.016, 0.008, and 0.004 mM. Untreated cells were used as negative controls. After continuous exposure for 96 h (at 37 °C, 5% CO_2_ and humidified atmosphere), the medium was replaced with 100 µL/well MTT solution (5 mg/mL MTT reagent in phosphate-buffered saline diluted 1:7 with fully supplemented DMEM), and plates were incubated for four hours. The MTT solution was removed, and the formed formazan crystals were dissolved in 100 µL DMSO per well. Optical densities were measured at 500 and 690 nm as measurement and reference wavelengths, respectively, with a microplate reader (HIDEX). The quantity of viable cells was expressed as a percentage of untreated controls. Every experiment was performed in triplicates and independently repeated three times. Statistical analysis was performed by GraphPad Prism 10.3.0. IC_50_ values were calculated via a sigmoidal interpolation (4PL, X is concentration).

#### 2.2.11. MRI Studies in Mice

All animal experiments in this study were conducted in accordance with a protocol approved by the Ethical Commission and Animal Research Commission at the Medical University of Vienna, following the Austrian Animal Welfare Law and related guidelines (project approval number: GZ 66.009/0371-V/3b/2018). Animals were allowed a two-week acclimation period prior to experimentation. Female athymic BALB/c mice, 4–6 weeks of age (Charles River), were used. Sixteen mice were randomly assigned to four groups (n = 4) and received intravenous injections of the experimental compound via the tail vein. Each mouse was administered 150 µL of the contrast agent dissolved in physiological NaCl, followed by a 50 µL NaCl flushing. The applied dose was selected based on in vitro relaxivity data and subsequent in vivo optimization. An initial concentration of 2 mM Mn was chosen by scaling the effective in vitro concentration (0.2 mM) to account for dilution upon intravenous administration. Dose-finding was then performed empirically by evaluating signal enhancement in relation to image quality, while considering injectability and tolerability. The final dose (0.67 mM Mn; 0.004 mmol/kg) was defined as the lowest concentration that provided robust vascular contrast enhancement while minimizing viscosity-related limitations and overall manganese exposure.

Dose optimization was guided by in vitro MRI relaxivity (r_1_) data, starting with a Mn concentration of 2 mM. The optimal dose was subsequently reduced to 0.67 mM Mn, corresponding to 0.004 mmol/kg body weight, which maintained effective signal enhancement in the mouse vasculature. Mice were euthanized after each MRI session via cervical dislocation approx. 120 min post-injection under inhalation anesthesia (2% isoflurane mixed with air), and organs were collected for biodistribution analysis.

## 3. Results

### 3.1. Synthesis and Physico-Chemical Characterization of CMD–DOTA–Mn(II), AD–DOTA–Mn(II), and CH–DOTA–Mn(II)

Covalent coupling strategies afforded three Mn(II)-chelating polysaccharide conjugates: CMD–DOTA–Mn(II); AD–DOTA–Mn(II); and CH–DOTA–Mn(II). The conjugation reactions were carried out in triplicate and proceeded with high reproducibility. Conjugation of CMD, AD, and CH to DOTA-NCS or DOTA-maleimide was accomplished under mild conditions, yielding up to 53% final product (as calculated after FPLC and lyophilization).

#### 3.1.1. Dynamic Light Scattering (DLS)

**Figure 1:**
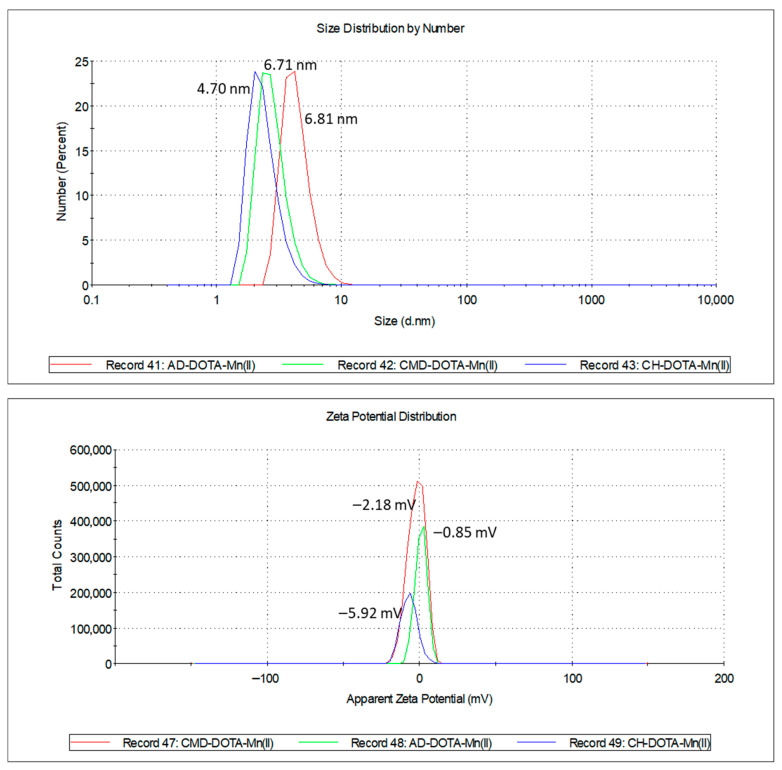
Hydrodynamic diameters (**top**) and zeta potentials (**bottom**) of CMD-DOTA-Mn(II), AD-DOTA-Mn(II), and CH-DOTA-Mn(II). Data represent average values from three independent measurements.

DLS analysis revealed hydrodynamic diameters in the nanoscale range. CMD-DOTA-Mn(II), AD-DOTA-Mn(II), and CH-DOTA-Mn(II) displayed sizes of 6.71, 6.81, and 4.70 nm, respectively, with modest negative zeta potentials ranging from −0.85 to −5.92 mV, indicating a near-neutral surface charge. These findings suggest that electrostatic repulsion is likely limited in these systems, and that additional stabilization mechanisms may contribute to the observed colloidal behavior (Figure 1, Table 1).

For the unmodified 70 kDa AD and 5 kDa CH (as provided by the manufacturer), polydisperse profiles have been observed. After attachment of the DOTA derivatives to AD, the size was 31.79 nm (±0.24 nm) and contracted after complexation with Mn(II) to 6.81 nm with a PDI = 0.3. The zeta potentials of the starting, intermediate, and final products of the 70 kDa AD were 34.6 mV, 1.92 mV, and −0.85 mV respectively (Table 1).

#### 3.1.2. ATR-FTIR of Modified Mn(II)-DOTA-Dextran Conjugates Was Performed to Confirm the Successful Coupling of the Macrocyclic Chelators to the Polysaccharide Molecule

**Figure 2:**
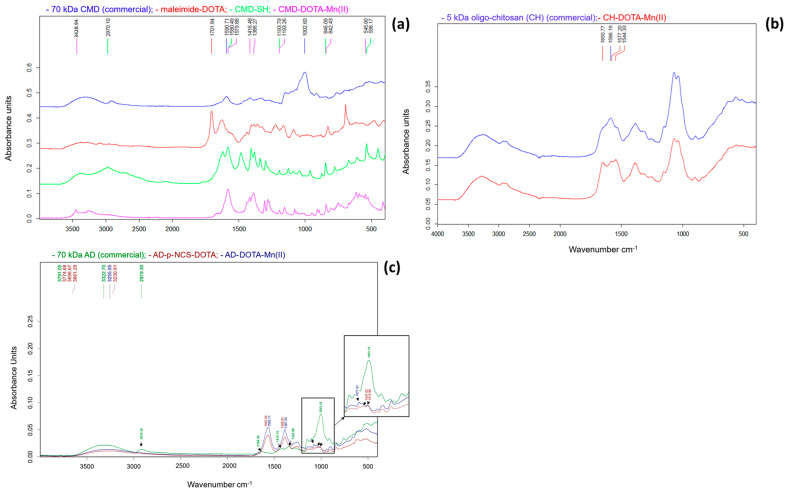
ATR-FTIR spectra of unmodified commercial poly-/oligosaccharides, intermediate DOTA-conjugates, and final Mn(II) complexes: (**a**) Commercial CMD (blue); intermediates CMD-SH; and CMD-DOTA (green and red curve); (**b**) 70 kDa aminodextran (AD) (green), AD-p-NCS-Bz-DOTA-GA conjugate (red), and the final AD-DOTA-Mn(II) (blue); (**c**) unmodified oligo-chitosan 5 kDa (CH) (blue) and the final CH-DOTA-Mn(II) (red).

The ATR-FTIR analysis validated the successful stepwise conjugations of Mn(II) to poly-/oligosaccharide conjugates. For CMD-SH, a broad band at 3375 cm^−1^ due to O–H stretching vibrations, and a band at 2970 cm^−1^ appeared, which refers to S–H stretching vibrations. The subsequent coupling with maleimide-DOTA introduced additional amide, aromatic, and O–H bending in aromatic ring signatures with characteristic peaks at 1580 cm^−1^, 1416 cm^−1^, and 1387 cm^−1^, respectively (Figure 2a). Upon Mn(II) complexation, subtle shifts in carboxylate and amide regions in the range of 1600–1300 cm^−1^ indicated the metal coordination (Figure 2a).

The spectrum of the starting 70 kDa AD showed absorption peaks at 3322 cm^−1^, 2919 cm^−1^, 1636 cm^−1^, and 1003 cm^−1^ assigned to O-H, C-H, C=O, and C-O stretching respectively (green spectrum on Figure 2b). The most prominent bands of the AD-p-NCS-Bz-DOTA-GA intermediate (red spectrum) were at 1565 cm^−1^, which resulted from C=O stretching vibrations, in the region from 1400 to 1200 cm^−1^ assigned to δ (C-H) and stretching vibrations of C-N and C-C in the region of 1013 cm^−1^, which is characteristic for C-O stretching vibration in the cycle. After coupling to p-NCS-Bz-DOTA-GA, the C-O band disappeared and was shifted and split into two bands at 1047 and 1013 cm^−1^, corresponding to DEX-p-NCS-Bz-DOTA-GA conjugation. Upon complexation of Mn(II) ions to AD-p-NCS-Bz-DOTA-GA, a strong absorption and shifting of the C=O band at 1565 cm^−1^ was seen and the C-O band was shifted to 1078 cm^−1^, indicating the coordination of the Mn(II) ion (blue spectrum) (Figure 2b). The FTIR spectrum of the final CH–DOTA–Mn(II) showed characteristic bands at 1651 cm^−1^, 1577 cm^−1^, and 1544 cm^−1^, which were attributed to C=O, C–N, and C–C stretching vibrations that were shifted upon complexation with Mn(II) compared to unmodified oligochitosan (Figure 2c).

#### 3.1.3. Isolation & Purification by Fast Protein Liquid Chromatography (FPLC)

**Figure 3:**
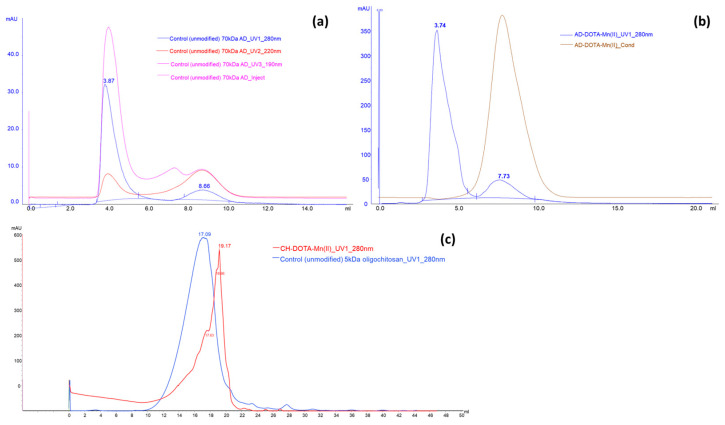
Selected chromatographic profiles of control (unmodified) AD 70 kDa (**a**), AD-DOTA-Mn(II) final conjugate (**b**), and unmodified CH (blue curve) and the final CH-DOTA-Mn(II) complex (red curve) (**c**).

FPLC purification enabled efficient removal of unreacted low-molecular-weight components (EDAC, cysteine, free DOTA derivatives, and MnCl_2_), resulting in well-defined conjugates. ICP-MS analysis confirmed Mn incorporation across all formulations, with Mn-to-polymer ratios consistent with the intended stoichiometries (Table 1). The two bigger constructs based on CMD and AD, using different covalent binding between the DOTA ligand and the sugar molecule showed comparable physiochemical properties. However, multiple chromatographic peaks were observed for AD-DOTA-Mn(II), suggesting partial heterogeneity or crosslinking during synthesis. Despite this, relaxivity values were reproducible across independent syntheses, reflecting the averaged behavior of the system. The observed heterogeneity may contribute to variations in molecular dynamics and hydration, which could influence physicochemical and biological properties. Two Sephadex G25 super-fine columns connected in a series were used for separation and isolation of CMD- and the AD- DOTA-Mn(II). The control-free CMD and 70 kDa AD eluted at 3.87 and at 3.82 mL, respectively. After a second purification step, a good isolation of the final AD-DOTA-Mn(II) (at 3.74 mL, main peak, with a peak tailoring at 7.73 mL) compound was observed (Figure 3). Unbound lower molecular compounds or reagents eluted after 12 mL, as monitored by the conductivity.

For the control, the free 70 kDa AD, one main peak was detected at 3.87 mL and a small tail peak at 8.66 (starting material as delivered by the manufacturer, Life Technologies/Molecular Probes, Grand Island, NY). The final AD-DOTA-Mn(II) product was collected at 3.74 mL and separated successfully from unreacted reagents (Figure 3b). For the CH-DOTA-Mn(II) compound with lower molecular weight, a Superdex 75 HR 10/30 column with an optimum separation range for polysaccharides 500–30 000 Da, was used as described in Section 2. The control 5 kDa CH eluted at 17.09 mL (main peak), detected by 280 nm. Discrete peak-fronting was observed after a coupling reaction of the modified DOTA molecule and after complexation with Mn(II), with small peaks detected at 17.63 and 18.86 mL, indicating small inter-population variations. The main peak of the final CH-DOTA-Mn was detected at 19.17 mL. This increased elution volume corresponds to the smaller hydrodynamic size of the final product.

#### 3.1.4. In Vitro MRI–Relaxivity (r_1_) Measurements

**Figure 4:**
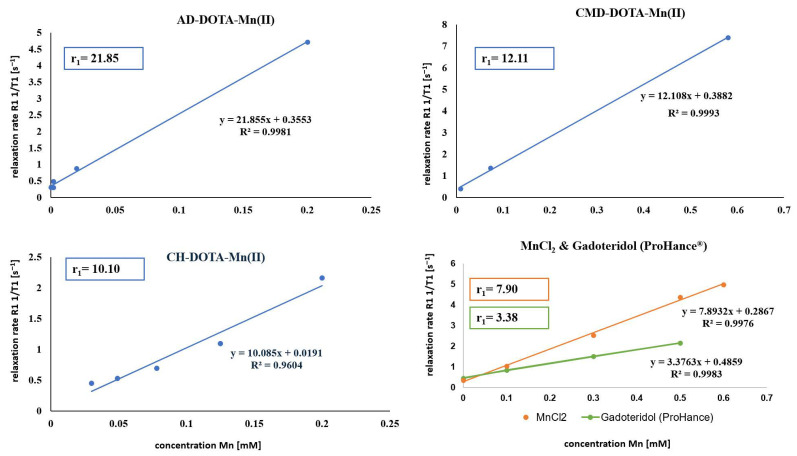
Plots of relaxation rates (R_1_, 1/T_1_, [s^−1^]) as a function of Mn concentration [mM] for AD-DOTA-Mn(II), CMD-DOTA-Mn(II), and CH-DOTA-Mn(II), compared to MnCl_2_ (red) and gadoteridol (ProHance^®^, green). R_1_ values were calculated from T_1_ measurements obtained by ROI-based analysis of phantom images, where mean T_1_ values and corresponding standard deviations (SD) were derived from signal distributions within each ROI. Error propagation from T_1_ to R_1_ (R_1_ = 1/T_1_) was performed using Gaussian error propagation. The resulting uncertainties in R_1_ were <1% and are therefore not visually distinguishable within the scale of the plot. A steep increase in the relaxation rates (R_1_) with r_1_ = 21.8, 12.1, and 10.1 for AD-DOTA-Mn(II), CMD-DOTA-Mn(II), and CH-DOTA-Mn(II), respectively, at 9.4 T was observed.

All three Mn(II)-tagged biopolymer formulations enhanced the longitudinal relaxivity (r_1_) compared with free MnCl_2_ and with clinically used Gadoteridol^®^ serving as a positive control. AD-DOTA-Mn(II) exhibited the highest relaxivity (r_1_ = 21.8 mM^−1^s^−1^), outperforming CMD-DOTA-Mn(II) (r_1_ = 12.1 mM^−1^s^−1^) and CH-DOTA-Mn(II) (r_1_ = 10.1 mM^−1^s^−1^) using a 9.4 T MR scanner (Figure 4).

**Figure 5:**
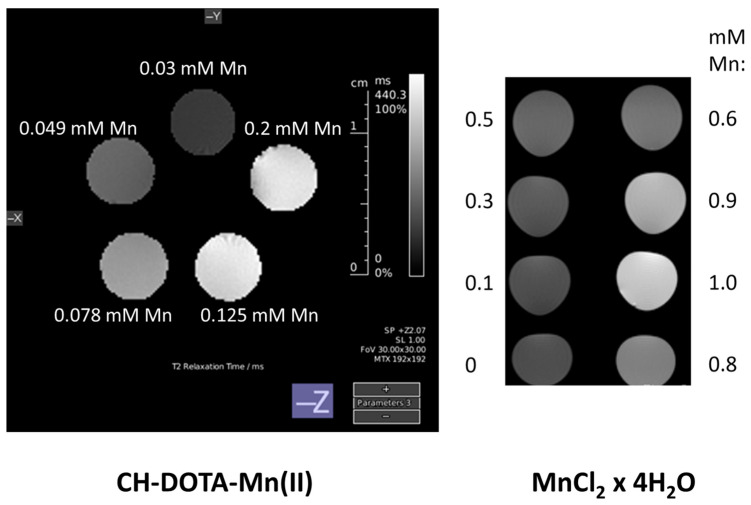
T2 weighted phantom MR images at 9.4 Tesla (RAREVTR TE = 7 ms TR 260–7500 in 12-step matrix: 128 × 128, FOV: 30 mm × 30 mm; slice: 1 mm). Cross-sections of self-made 1 mL-syringe-tubes filled with CH-DOTA-Mn(II) (concentrations from 0.03 to 0.2 mM) and dilution series of MnCl_2_ × 4H_2_O (concentration range from 0.0 to 1.0 mM) as a positive control. Note the strong signal enhancement coming from the 0.2 mM sample compared to the 1 mM positive control (MnCl_2_ × 4H_2_O).

The enhanced relaxometric performance was further confirmed by the corresponding phantom MR images (Figure 5). Notably, CH-DOTA-Mn(II) produced pronounced signal enhancement already at 0.2 mM, exceeding the signal intensity of the MnCl_2_ control even at a higher concentration (1 mM). This behavior can be attributed to the macromolecular nature of the conjugates, which reduces rotational mobility (τ_R_) and thereby enhances proton relaxation efficiency.

#### 3.1.5. Short-Term Physicochemical and Relaxometric Stability

To assess the short-term stability of the macromolecular Mn(II) complexes, CMD–DOTA–Mn(II) and CH–DOTA–Mn(II) were evaluated under physiologically relevant conditions (0.9% NaCl) over a period of seven days at 4 °C (in the dark) and at room temperature (RT).

Both formulations remained visually clear and transparent throughout the incubation period, with no evidence of turbidity or precipitation (Figure S1), indicating excellent colloidal stability under ionic conditions. This behavior is consistent with effective steric stabilization provided by the hydrated polysaccharide shell.

Dynamic light scattering (DLS) analysis revealed only minor changes in hydrodynamic diameter after seven days of storage at RT. CMD–DOTA–Mn(II) exhibited a marginal size increase of approximately 4% (6.70 ± 0.013 nm to 7.02 ± 0.019 nm), while CH–DOTA–Mn(II) showed a slightly larger increase of approximately 11% (4.79 ± 0.050 nm to 5.30 ± 0.840 nm). These variations are not indicative of aggregation but are more likely attributable to minor conformational rearrangements, hydration effects, or slight swelling of the polymer matrix.

Importantly, no additional size populations or aggregation-related features were observed, supporting the maintenance of colloidal integrity.

Relaxometric analysis further confirmed the functional stability of the complexes. Only minimal variations in longitudinal relaxivity (r_1_) were observed after seven days under both storage conditions. CMD–DOTA–Mn(II) showed a slight increase in r_1_ (11.75 to 12.36 mM^−1^·s^−1^), while CH–DOTA–Mn(II) remained essentially unchanged (12.14 to 12.11 mM^−1^·s^−1^). These findings indicate preservation of the Mn(II) coordination environment and absence of significant demetallation or structural degradation.

### 3.2. Effect on Cell Viability

**Figure 6:**
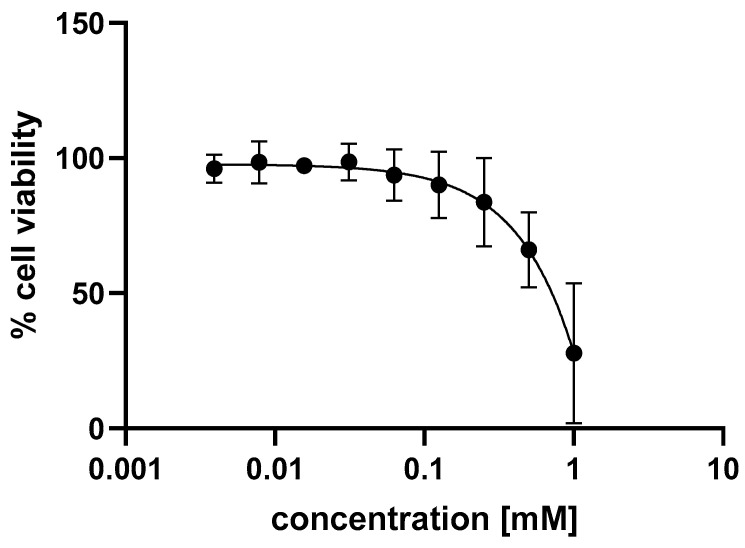
Cell viability assessment of mouse fibroblast NIH-3T3 exposed to CH-DOTA-Mn(II) over 96 h.

The MTT assay revealed a dose-dependent cytotoxicity of CH-DOTA-Mn(II) in NIH-3T3 fibroblasts. Cell viability remained above 80% at concentrations ≤ 0.125 mM, while IC_50_ was determined at approximately 0.75 mM after 96 h exposure (Figure 6). These results suggest that the smaller CH conjugate may exert more pronounced cellular interactions due to its higher diffusivity and potential intracellular uptake. By contrast, higher-molecular-weight CMD and AD conjugates are expected to exhibit reduced cellular penetration, thereby lowering direct cytotoxic risks. In sum, the continuous exposure of CH-DOTA-Mn(II) to healthy NIH-3T3 cells over a period of 96 h performed in the concentration range of 0.004–0.125 mM showed no significant effect of this experimental compound on cell viability. The IC_50_ was calculated as 755 ± 249 µM.

### 3.3. Biodistribution of CMD-, AD-, and CH-DOTA-Mn(II) by ICP-MS

ICP–MS analysis revealed distinct organ-specific biodistribution profiles for the three DOTA–Mn(II) conjugates in mice (Table 2). CH-DOTA-Mn(II) exhibited the highest manganese accumulation among the tested compounds, particularly in the liver and kidneys. At the highest dose (1 mM Mn), mean Mn concentrations reached 20.57 mg/kg in the liver and 6.68–6.82 mg/kg in the kidneys. This pronounced hepatic and renal uptake suggests predominant hepatobiliary and renal clearance, consistent with the low molecular weight (5 kDa) of the CH carrier, which facilitates glomerular filtration and subsequent excretion.

### 3.4. In Ovo MRI

**Figure 7:**
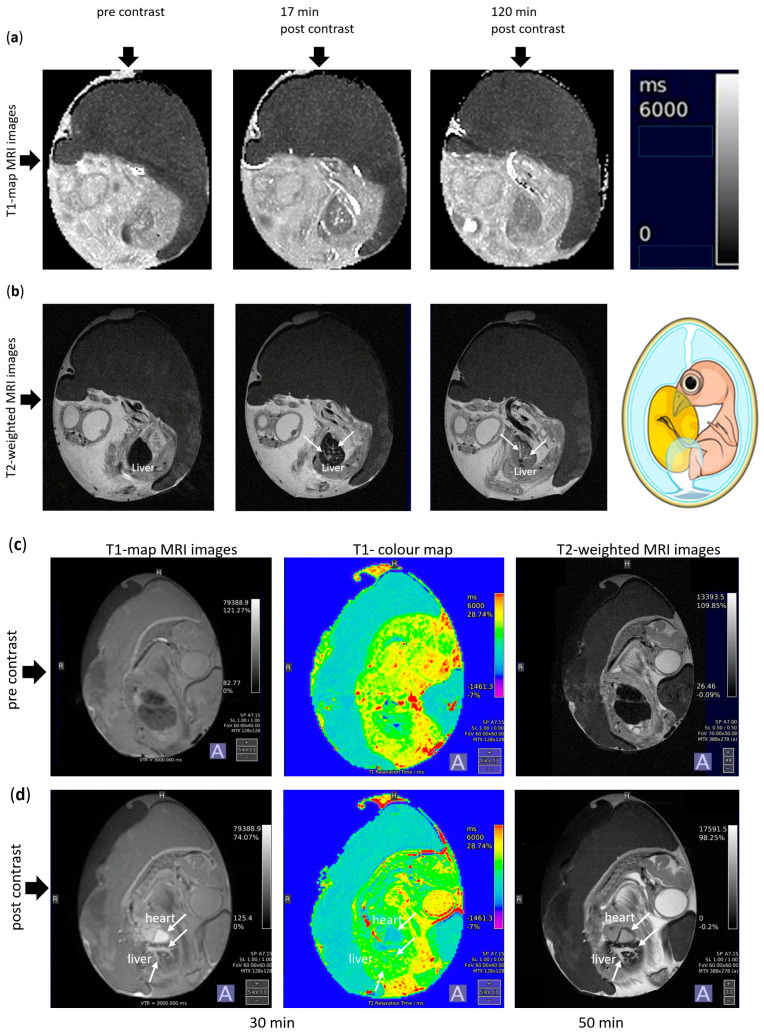
MRI images: (**a**) A RARE VTR sequence was used to evaluate the T1 changes using CMD-DOTA-Mn(II) as a contrast agent. A strong signal enhancement was visible 17 min after injection, particularly in the blood vessels of the liver (white arrows); (**b**) To evaluate changes in T2 relaxation, a T2-weighted RARE sequence was applied. A strong enhancement of the vasculature was seen after 17 min and after 120 min throughout the liver. Slice orientation is slightly different between the time points because of the manipulation (injection, incubation) between the MR measurements. Analogously, signal enhancement was observed with CH-DOTA-Mn(II) after 30 min and 50 min post injection, highlighting the blood vessels of the heart and the liver of the chicken embryo (MRI images (**c**,**d**)).

The in ovo model enabled visualization and initial screening of biodistribution patterns. CMD-DOTA-Mn(II) (Figure 7a,b) generated strong vascular enhancement persisting up to 120 min post-application, while CH-DOTA-Mn(II) showed a faster distribution and accumulation of the contrast agent up to 50 min post-injection (Figure 7c,d), consistent with its lower molecular weight. These findings corroborate the size-dependent pharmacokinetics anticipated from the conjugation strategy. Representative MR images with CMD-DOTA-Mn(II) and CH-DOTA-Mn(II) are shown in Figure 7. No signal enhancement was observed with the AD-DOTA-Mn(II) at the applied concentration of 0.07 mM Mn. Dose-increasing led to increased viscosity and adverse effects with this compound.

### 3.5. MRI in Nude Mice

**Figure 8:**
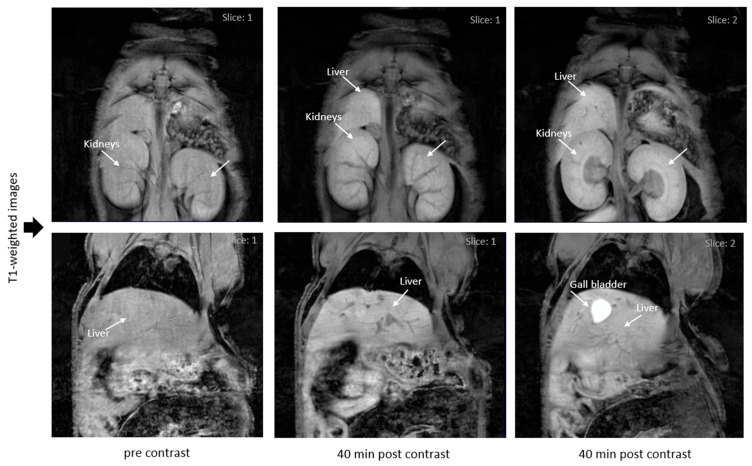
In vivo MRI images with CH-DOTA-Mn(II): A T1-weighted FLASH sequence was used to evaluate the T1 changes. A strong signal enhancement was visible in the kidneys, liver, and the gall bladder, which persisted at least 40 min post injection.

Dose-finding studies were performed for all three sugar-based Mn(II) complexes. However, CMD-DOTA-Mn(II) and AD-DOTA-Mn(II) exhibited high viscosity at the concentrations required for effective signal, which limited injectability and precluded further in vivo evaluation at higher concentrations. In contrast, CH-DOTA-Mn(II) demonstrated favorable handling properties and in vivo performance. A dose corresponding to 0.67 mM Mn (0.004 mmol/kg body weight) was sufficient to achieve robust vascular contrast enhancement in nude mice (Figure 8) while limiting systemic manganese exposure.

## 4. Discussion

Covalent grafting of DOTA derivatives onto polysaccharide backbones generated stable macrocyclic coordination sites for Mn(II). Maleimide-DOTA-GA was coupled to CMD via thiol-maleimide chemistry (Scheme 1), while p-NCS-Bz-DOTA-GA enabled amide bond formation with primary amines of AD and CH (Scheme 2). ATR-FTIR and FPLC confirmed successful conjugation and structural integrity prior to metal coupling.

Manganese incorporation in the present systems was achieved by complexation of MnCl_2_ under mild aqueous conditions using the DOTA-type macrocyclic ligands. Based on the established coordination chemistry of MRI-relevant manganese chelates, Mn(II) was the expected oxidation state in the resulting conjugates. Numerous studies have emphasized that macrocyclic ligand development in this field is primarily directed toward Mn(II) stabilization, whereas Mn(III) is considerably more difficult to maintain in aqueous and physiological environments because of its tendency toward hydrolysis and disproportionation [20,21,22]. Recent works introducing cryogenic X-ray photoelectron spectroscopy (cryo-XPS) for nanomedicines highlight the value of frozen-hydrated approaches for soft nanosystems [23,24]. Thus, future studies that include cryo-XPS in combination with complementary methods such as electron paramagnetic resonance (EPR) will be used to further validate manganese speciation.

DLS analysis revealed hydrodynamic diameters in the nanoscale range. CMD-DOTA-Mn(II), AD-DOTA-Mn(II), and CH-DOTA-Mn(II) displayed sizes of 6.71, 6.81, and 4.70 nm, respectively, with modest negative zeta potentials ranging from −0.85 to −5.92 mV, indicating an overall near-neutral surface charge. At first glance, such low absolute ζ-potential values could be interpreted as indicative of limited electrostatic stabilization. However, polysaccharide-based systems are well known to exhibit stable colloidal behavior despite near-neutral surface charge due to dominant steric effects, as reported for dextran- and polymer-coated materials [25,26,27]. In the present study, colloidal stability is therefore attributed primarily to steric stabilization that arose from the hydrated polysaccharide shell. The highly solvated carbohydrate chains form an extended hydration layer that generates an entropic and osmotic barrier, effectively limiting particle–particle interactions and aggregation even in the absence of significant electrostatic repulsion. Accordingly, the observed combination of small hydrodynamic size and near-neutral ζ-potential is not indicative of instability but rather consistent with a sterically stabilized, well-dispersed system. These features are considered favorable for maintaining dispersion stability in biological environments and may support prolonged circulation behavior (Figure 1, Table 1). In addition, CMD–DOTA–Mn(II) and CH–DOTA–Mn(II) remained colloidally stable over seven days in saline, with no evidence of aggregation or precipitation. Only minor increases in hydrodynamic size were observed, which are consistent with subtle conformational or hydration-related changes rather than instability.

Importantly, the relaxometric properties remained essentially unchanged over time, indicating preservation of the Mn(II) coordination environment and absence of significant demetallation. This observation provides indirect but functionally highly relevant evidence of kinetic stability in the absence of classical thermodynamic stability constants, which are difficult to define for polydisperse macromolecular systems.

To further elucidate the structural properties of the conjugates, high-resolution transmission electron microscopy (HRTEM) and elemental mapping techniques were considered. However, the investigated systems consist of highly hydrated, soft macromolecular assemblies with hydrodynamic diameters of approximately 5–7 nm. For such polymeric systems, conventional TEM/HRTEM imaging is associated with inherent limitations, as sample preparation typically involves solvent removal and imaging under high vacuum, which may alter the native hydrated structure, reduce electron density contrast, and induce structural deformation or collapse. As a result, the obtained images may not reliably represent the true morphology or nanoscale organization of the conjugates. These challenges have been widely discussed for soft and polymer-coated nanomaterials, where discrepancies between solution-state techniques, e.g., DLS, and electron microscopy often arise due to loss of the hydrated polymer shell and preparation-induced artifacts [28,29,30].

Accordingly, HRTEM and EDS mapping were not pursued in the present study. Future investigations will focus on advanced approaches such as cryogenic transmission electron microscopy (cryo-TEM) and scanning transmission electron microscopy combined with energy-dispersive X-ray spectroscopy (STEM-EDS), which are better suited for preserving the native hydrated state of soft nanomaterials and enabling a more reliable assessment of Mn distribution.

Manganese-based MRI contrast agents have reemerged as alternatives to Gd formulations due to favorable paramagnetic properties and the absence of Gd(III)-associated retention concerns. Although MnCl_2_ (LumenHance^®^) [31] and MnDPDP (Teslascan^®^) [32] achieved regulatory approval, broader use was limited by rapid clearance and safety concerns related to Mn release at high or repeated doses [33]. These limitations emphasize the need for Mn complexes that combine high thermodynamic stability, kinetic inertness, and improved relaxometric efficiency.

From a coordination chemistry perspective, DOTA-type macrocyclic ligands effectively saturate the coordination sphere of Mn(II), thereby preventing the presence of inner-sphere water molecules. Consequently, the observed relaxivity enhancement cannot be attributed to classical inner-sphere mechanisms. Instead, it arises from a combination of second- and outer-sphere contributions. In particular, hydrogen-bonded water molecules in the vicinity of the coordinated complex can participate in second-sphere interactions, while outer-sphere relaxation is governed by the diffusion of bulk water near the paramagnetic center. In addition, conjugation to polysaccharide backbones reduces rotational mobility (τ_R_), which enhances dipole–dipole interactions and thereby increases relaxivity. Within the present constructs, the increased r_1_ values are therefore best explained by the combined effect of slowed molecular tumbling and enhanced second-sphere hydration within the hydrated polymer matrix, in agreement with established relaxivity theory and recent reports on Mn-based macrocyclic and macromolecular contrast agents [1,20,21,22]. Various Mn-based macromolecular systems—including dendritic chelates [34,35] and nanoparticle platforms [36,37,38,39]—have demonstrated enhanced relaxivity through modulation of molecular architecture [1]. Consistent with this concept, all sugar-based conjugates prepared in this study exhibited r_1_ values of 10.1–21.8 mM^−1^s^−1^, exceeding MnCl_2_ (7.2 mM^−1^s^−1^) and gadoteridol (3.4 mM^−1^s^−1^) at 9.4 T. Because DOTA-type ligands saturate the primary coordination sphere of Mn(II), the observed relaxivity enhancement is unlikely to originate from inner-sphere water exchange. Instead, it is most plausibly attributed to reduced rotational mobility and increased second-sphere/outer-sphere water interactions within the hydrated polysaccharide matrix. Comparable effects have been reported for polysaccharide- and protein-based Mn systems [40,41,42].

Although Mn loading differed among constructs (approximately 50, 43, and 20 Mn per carrier for AD, CMD, and CH, respectively), relaxivity reflects a combination of metal content, molecular size, hydration, and dynamic behavior rather than metal density alone. Importantly, the different coupling strategies did not measurably alter molar relaxivity, indicating comparable Mn coordination environments. FPLC revealed partial heterogeneity in AD conjugates, suggesting species of varying substitution or hydrodynamic size. These data are most likely attributable to a combination of factors, including variable degrees of DOTA substitution, partial crosslinking during the conjugation process, and conformational variability of the polymer backbone. From a physicochemical perspective, such heterogeneity may influence key parameters such as molecular tumbling rates and local hydration environments, which are known to affect relaxometric properties. From a translational standpoint, the observed heterogeneity may also contribute to differences in viscosity and pharmacokinetic behavior, consistent with the limitations encountered during in vivo evaluation. These findings highlight the importance of improved control over substitution patterns and structural uniformity in future studies to further enhance reproducibility and facilitate interpretation of structure-function relationships. Such heterogeneity may enhance relaxivity by further slowing rotational motion, but it also highlights the importance of batch control and structural definition.

Translationally, formulation properties proved decisive. CMD- and AD-based conjugates exhibited increased viscosity at concentrations required to achieve an effective MRI signal (≥1 mM Mn equivalent), limiting injectability despite favorable relaxivity. This highlights an inherent balance between molecular size, relaxivity, and injectability in macromolecular contrast agents. While larger polymer architectures can enhance relaxivity through reduced rotational mobility, they may simultaneously impair injectability due to increased viscosity and altered flow behavior, particularly at higher concentrations. In addition, polymer architecture and degree of substitution are expected to influence solution viscosity and flow behavior, thereby affecting handling and in vivo applicability. These findings emphasize the importance of balancing physicochemical and functional properties during probe design. Future optimization strategies may include reducing polymer molecular weight, fine-tuning the degree of substitution, and applying dilution or co-solvent approaches to mitigate viscosity-related limitations.

In contrast, CH–DOTA–Mn(II) provided enhanced relaxivity with superior handling characteristics, supporting its prioritization for further development.

In addition, CH–DOTA–Mn(II) demonstrated the most favorable physicochemical properties, including lower molecular weight, improved injectability, and superior in vivo performance. Therefore, cytotoxicity evaluation in the present study was focused on this formulation. While the higher-molecular-weight CMD- and AD-based conjugates are generally expected to exhibit reduced cellular uptake and, consequently, lower cytotoxicity—consistent with established trends for macromolecular systems—no direct cytotoxicity data are available for these formulations within the scope of this study. Future investigations will include a systematic cytotoxicity assessment of all conjugates to enable a comprehensive evaluation of their safety profiles.

Biodistribution studies showed predominant hepatic and renal manganese accumulation across all formulations, consistent with hepatobiliary and renal elimination pathways. Higher renal levels for CH–DOTA–Mn(II) are consistent with its smaller hydrodynamic size and partial glomerular filtration. Extrahepatic tissues exhibited substantially lower Mn concentrations. Brain levels were low relative to clearance organs at the investigated time point. While detectable, they do not permit definitive conclusions regarding blood–brain barrier permeability without baseline-controlled or time-resolved analyses.

From a coordination chemistry perspective, Mn(II) relaxivity arises from its high-spin d^5^ electronic configuration and favorable electronic relaxation properties [43]. Macrocyclic DOTA-type ligands form thermodynamically stable coordination cages around Mn(II) [44,45], and slowing rotational motion through macromolecular conjugation remains a principal strategy for maximizing r_1_ in both Mn(II) and Gd(III) systems [46].

Importantly, increasing regulatory scrutiny regarding long-term gadolinium retention in brain and peripheral tissues has intensified efforts to develop non-gadolinium contrast agents with well-defined stability and clearance profiles. Within this context, Mn(II)–DOTA macromolecular systems offer a chemically rational platform that combines macrocyclic stability with relaxivity enhancement through controlled modulation of molecular dynamics.

Overall, polysaccharide-conjugated Mn(II)–DOTA complexes achieve enhanced relaxivity primarily via macromolecular control of rotational dynamics while maintaining physiologically consistent clearance patterns. Among the investigated constructs, CH–DOTA–Mn(II) provided the most balanced profile of relaxivity, injectability, and in vivo behavior. Further studies focusing on kinetic stability, transmetallation resistance, pharmacokinetics, and long-term safety are warranted to define translational potential.

## 5. Conclusions

The present findings demonstrate that Mn(II) chelates of sugar-based biopolymers can be synthesized reproducibly, exhibiting favorable imaging properties and providing effective vascular contrast in vivo. Beyond their high Mn^2+^ loading capacity, these conjugates offer tunable circulation profiles determined by their molecular weight, and they display inherent biodegradability. Among the tested formulations, CMD- and AD-DOTA-Mn(II) may be applied as blood pool agents for MRI, but further dose-finding studies are needed. In contrast, the lower molecular weight CH-DOTA-Mn(II) suggests utility in applications where fast renal elimination is desirable. Moreover, these data support the continued development of Mn-based poly-/oligosaccharide conjugates as promising and potentially safer alternatives to GBCA. Short-term stability studies further demonstrated that the investigated Mn(II) conjugates maintain their physicochemical integrity and relaxometric performance under physiologically relevant conditions, supporting their potential for preclinical and translational development. Future investigations should prioritize rigorous long-term stability and safety assessments, optimization of Mn loading density, and evaluation in pathological models with altered vascular permeability to fully elucidate their clinical potential.

## Figures and Tables

**Table 1:** Comprehensive overview of the prepared CMD-DOTA-Mn(II), AD-DOTA-Mn(II), and CH-DOTA-Mn(II) conjugates and their physico-chemical properties. Parameters include Mn/poly-oligosaccharide starting concentrations, longitudinal relaxivity (r_1_), hydrodynamic size, ζ-potential, and molar ratios. Relaxivity values were compared with the gadoteridol (ProHance^®^) in clinical use, and with MnCl_2_ as positive controls, all measured in PBS at 9.4 T.

Conjugate	Mn/Polymer Conc. (mM)	r_1_ (mM^−1^·s^−1^)	r_1_ (mM^−1^·s^−1^), Published Elsewhere	Hydrodynamic Size (nm) ^a^	ζ-Potential (mV) ^a^	Mn/Polymer Molar Ratio
CMD-DOTA-Mn(II)	0.3/0.007	12.11	—	6.71 ± 0.013	−2.18 ± 0.44	43
AD-DOTA-Mn(II)	0.5/0.01	21.82	—	6.81 ± 0.014	−0.85 ± 0.53	50
CH-DOTA-Mn(II)	0.8/0.04	10.1	—	4.70 ± 0.013	−5.92 ± 0.71	20
Gadoteridol (ProHance^®^)	—	3.4	3.7 [18]	—	—	—
MnCl_2_	—	7.2	8.0 [19]	—	—	—

^a^ Data represent average values from three independent measurements. Experimental conditions: viscosity = 0.8872 cP; refractive index (RI) = 1.330.

**Table 2:** Concentrations of manganese (mg Mn/kg tissue) in the brain, kidneys (right, r; left, l), heart, spleen, lungs, and liver of treated mice. Data are expressed as mean ± SD (n = 4).

Compound/Concentration	Brain (mg/kg)	Kidneys r/L (mg/kg)	Heart (mg/kg)	Spleen (mg/kg)	Lungs (mg/kg)	Liver (mg/kg)
CMD-DOTA-Mn(II)/1 mM Mn	0.227 ± 0.0025	0.981 ± 0.0010/1.024 ± 0.0022	1.854 ± 0.068	0.149 ± 0.0019	0.202 ± 0.0016	3.582 ± 0.0023
AD-DOTA-Mn(II)/1 mM Mn	0.362 ± 0.0011	2.819 ± 0.0012/2.997 ± 0.0028	1.050 ± 0.014	0.315 ± 0.0043	0.149 ± 0.0028	4.618 ± 0.0018
CH-DOTA-Mn(II)/1 mM Mn	0.490 ± 0.0015	6.679 ± 0.0020 /6.820 ± 0.0010	2.336 ± 0.012	0.548 ± 0.0024	1.246 ± 0.0016	20.57 ± 0.0019
CH-DOTA-Mn(II)/0.67 mM Mn	0.446 ± 0.0083	5.418 ± 0.0041/6.296 ± 0.0041	1.188 ± 0.072	0.481 ± 0.0023	0.555 ± 0.0047	9.53 ± 0.0019

## Data Availability

Additional data related to this article can be found online at https://data.mendeley.com/datasets/zcsdjh7cwm (accessed on 15 April 2026).

## Supplementary Materials

The following supporting information can be downloaded at: https://www.mdpi.com/article/10.3390/pharmaceutics18050530/s1, Figure S1: Photographs of CMD-DOTA-Mn(II) and CH-DOTA-Mn(II) dissolved in aqueous physiological NaCl solution after incubation at 4 °C and at room temperature for seven days; Figure S2: Average hydrodynamic diameters (daver.) of CMD-DOTA-Mn(II) (A) and CH-DOTA-Mn(II) (B) at day 0 and 7 days after incubation at RT; Figure S3: Plots of relaxation rates (R_1_, [s^−1^]) as a function of Mn concentration [mM] for CMD-DOTA-Mn(II) (first row) and CH-DOTA-Mn(II) (second row) after 7 days storage at 4 °C in dark and at room temperature (RT), no exclusion of light; Table S1: Longitudinal relaxivities (r_1_) and average hydro-dynamic diameter of CMD-DOTA-Mn(II) and CH-DOTA-Mn(II) conjugates.

## Abbreviations

The following abbreviations are used in this manuscript:

AD	Aminodextran
ATR–FTIR	Attenuated Total Reflectance Fourier-Transform Infrared Spectroscopy
BBB	Blood–Brain Barrier
CAM	Chorioallantoic Membrane
CA	Contrast Agent
CMD	Carboxymethyldextran
DLS	Dynamic Light Scattering
DMSO	Dimethyl Sulfoxide
DOTA	1,4,7,10-Tetraazacyclododecane-1,4,7,10-tetraacetic Acid
EDAC	1-Ethyl-3-(3-dimethylaminopropyl)carbodiimide
FPLC	Fast Protein Liquid Chromatography
GBCA	Gadolinium-Based Contrast Agent
ICP–MS	Inductively Coupled Plasma–Mass Spectrometry
MEMRI	Manganese-Enhanced Magnetic Resonance Imaging
Mn	Manganese
MRI	Magnetic Resonance Imaging
MTT	3-(4,5-Dimethylthiazol-2-yl)-2,5-diphenyltetrazolium Bromide
NSF	Nephrogenic Systemic Fibrosis
PBS	Phosphate-Buffered Saline
PDI	Polydispersity Index
ROI	Region of Interest
SNR	Signal-to-Noise Ratio
