# Supplementary material for: Mn(II)-Tagged DOTA-Modified Sugar-Based Biopolymers as Gadolinium-Free Contrast Agents for Magnetic Resonance Imaging
Source: Pharmaceutics. 2026 Apr 27;18(5):530. doi: 10.3390/pharmaceutics18050530 (PMC13210614; doi:10.3390/pharmaceutics18050530)
Supplement: Supplementary file 1 [file pharmaceutics-18-00530-s001.zip › pharmaceutics-4214538-supplementary.pdf]

pharmaceutics

Mn(II)-Tagged DOTA-Modified Sugar-Based Biopolymers as Gadolinium-Free Contrast Agents for Magnetic Resonance Imaging

Irena Pashkunova-Martic [1,2,*], Joachim Friske [1], Silvester J. Bartsch [1,3], Daniela Prinz [1], Theresa Balber [4,5], Verena Pichler [5,6,7], Dieter Baurecht [8], Bernhard K. Keppler [2] and Thomas H. Helbich [1]

1 Department of Biomedical Imaging and Image-Guided Therapy, Division of Molecular and Structural Preclinical Imaging, Medical University of Vienna & General Hospital of Vienna , Waehringer Guertel 18-20, 1090 Vienna, Austria; joachim.friske@meduniwien.ac.at (J.F.); silvester.bartsch@tum.de (S.J.B.); daniela.a.prinz@meduniwien.ac.at (D.P.); thomas.helbich@meduniwien.ac.at (T.H.H.)

2 Institute of Inorganic Chemistry, Faculty of Chemistry, University of Vienna, Waehringer Strasse 42, 1090 Vienna, Austria; bernhard.keppler@univie.ac.at

3 Department of Nuclear Medicine, TUM Klinikum Rechts Der Isar, Technical University of Munich, 80333 Munich , Germany

4 Department of Biomedical Imaging and Image-Guided Therapy, Division of Nuclear Medicine, Medical University of Vienna & General Hospital of Vienna, Waehringer Guertel 18–20, 1090 Vienna, Austria; theresa.balber@meduniwien.ac.at

5 Joint Applied Medicinal Radiochemistry Facility, University of Vienna and Medical University of Vien-na , 1090 Vienna, Austria; verena.pichler@univie.ac.at

6 Department of Pharmaceutical Sciences, University of Vienna, Josef-Holaubek-Platz 2 (UZA II), 1090 Vienna, Austria

7 Department of Pharmacy, University of Oslo , Problemveien 11, 0313 Oslo, Norway

8 Department of Physical Chemistry, Faculty of Chemistry, University of Vienna, Waehringer Strasse 42, 1090 Vienna, Austria; dieter.baurecht@univie.ac.at

* Correspondence: irena.pashkunova-martic@meduniwien.ac.at; Tel./Fax: +43- 1-40400-48190

Stability Assessment of Mn(II)-DOTA–Polysaccharide Conjugates

The stability of a metal chelate is a critical parameter for its subsequent in vivo application. In principle, determination of the thermodynamic stability constant (log K) of a metal complex requires well-defined protonation constants of the corresponding ligand. However, due to the presence of chemically non-equivalent amine and carboxylate sites, as well as the intrinsic molecular weight distribution of polymeric carriers such as carboxymethyldextran (CMD) and oligochitosan (CH), discrete and accurately defined protonation constants cannot be reliably established. Consequently, a rigorous determination of thermodynamic stability constants is not feasible for these macromolecular Mn(II) chelates.

 https://doi.org/10.3390/pharmaceutics18050530

Instead, complementary experimental approaches were employed to assess their physicochemical and functional stability under physiologically relevant conditions. Specifically, the colloidal stability of CMD–DOTA–Mn(II) and CH–DOTA–Mn(II) was evaluated in physiological saline (0.9% NaCl) after incubation for seven days at 4 °C (in the dark) and at room temperature (RT). Both formulations remained clear and transparent throughout the incubation period, with no visible turbidity or precipitation, indicating excellent colloidal stability (Figure S1). This observation suggests that the hydrated polysaccharide shell provides effective steric stabilization, preventing aggregation even under ionic conditions that would typically screen electrostatic repulsion.

https://doi.org/10.3390/pharmaceutics18050530

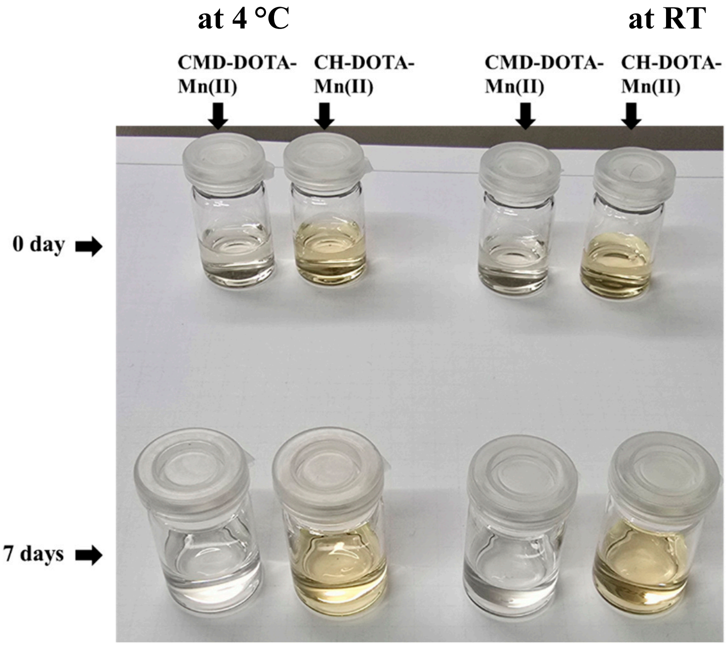

Figure S1. Photographs of CMD-DOTA-Mn(II) and CH-DOTA-Mn(II) dissolved in aqueous physiological NaCl solution after incubation at 4 °C and at room temperature for seven days.

DLS Stability Analysis

Dynamic light scattering (DLS) measurements were performed to monitor potential changes in hydrodynamic size over time (Figure S2).

After seven days of storage at RT:

- CMD–DOTA–Mn(II) exhibited a minor increase in hydrodynamic diameter (~4%)
- CH–DOTA–Mn(II) showed a slightly larger increase (~11%)

These modest changes are not indicative of aggregation but are more likely attributable to:

- minor conformational rearrangements of the hydrated polymer chains,
- slight swelling effects due to prolonged solvent exposure,
- and/or changes in the hydration shell.

Importantly, no evidence of particle aggregation (e.g., large size populations or visible precipitation) was observed, supporting the conclusion of maintained colloidal stability.

A. DLS of CMD-DOTA-Mn(II)

https://doi.org/10.3390/pharmaceutics18050530

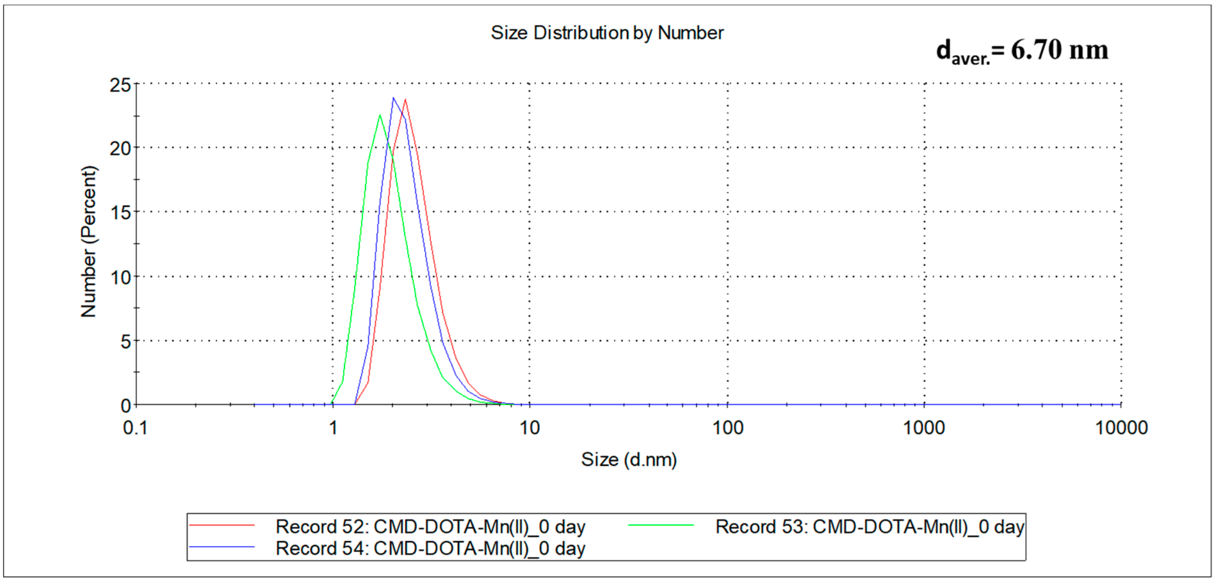

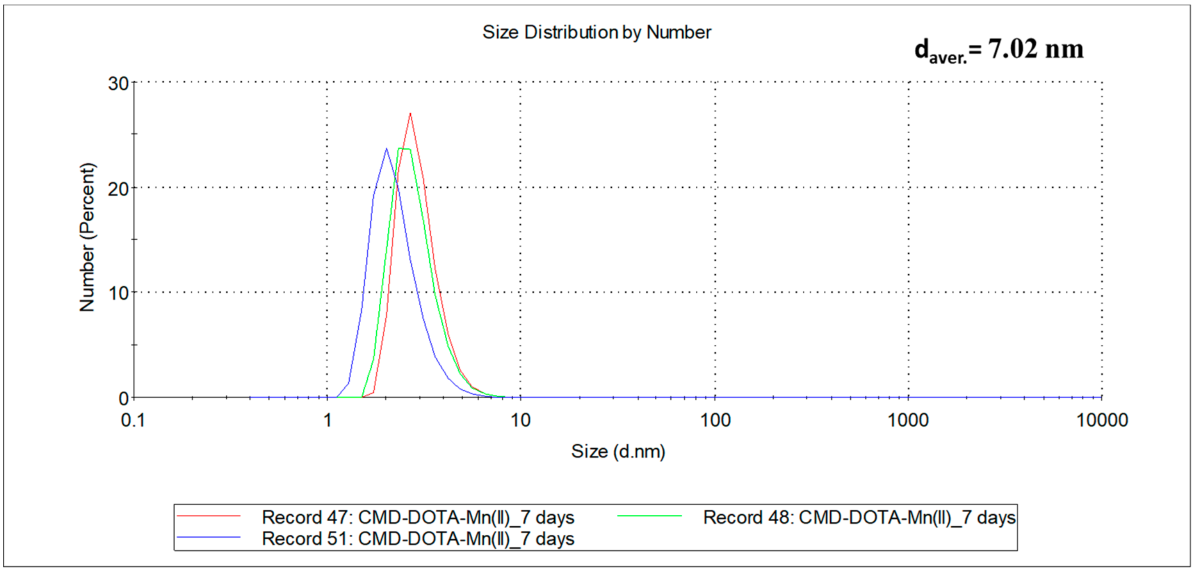

B. DLS of CH-DOTA-Mn(II)

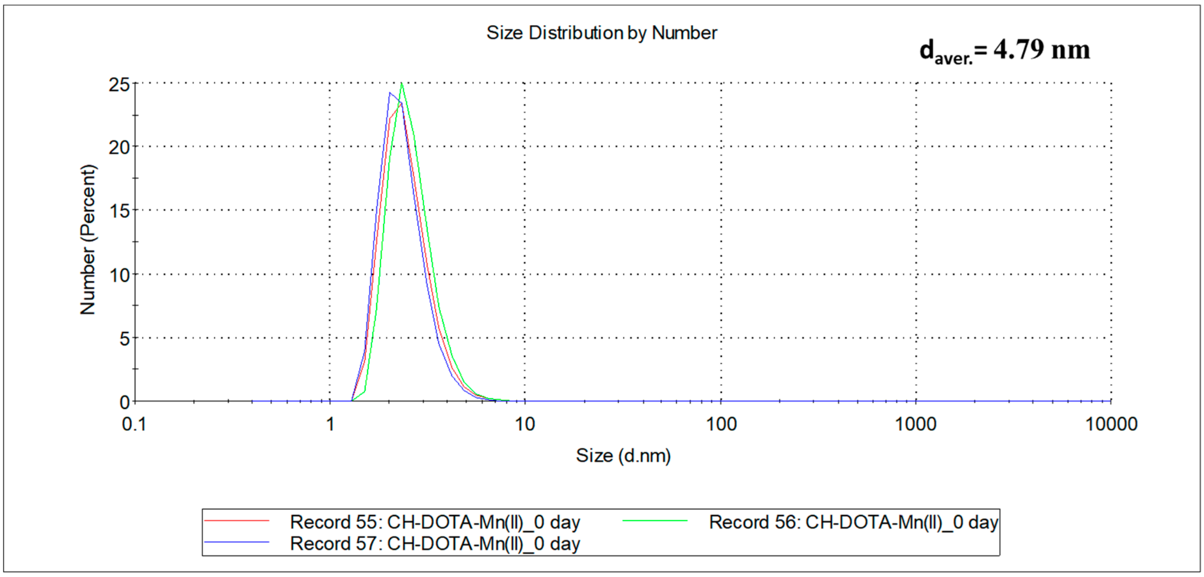

https://doi.org/10.3390/pharmaceutics18050530

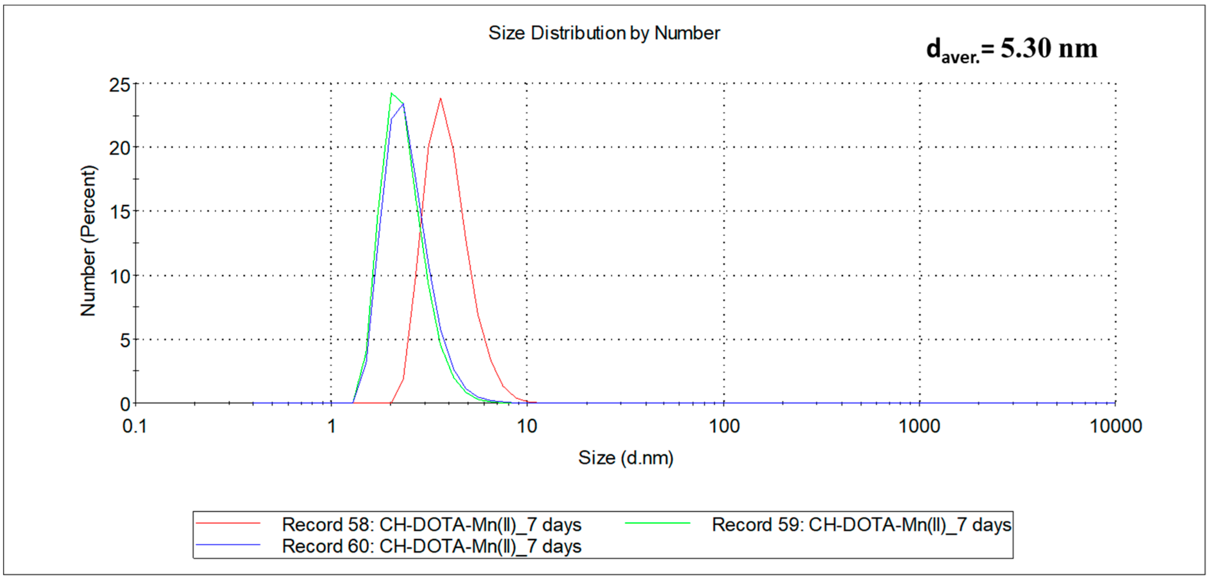

Figure S2. Average hydrodynamic diameters ($d_{aver.}$) of CMD-DOTA-Mn(II) (**A.**) and CH-DOTA-Mn(II) (**B.**) at day 0 and 7 days after incubation at RT. Data represent average values from three independent measurements.

Relaxometric Stability

To further assess functional stability, changes in longitudinal relaxation rates (R_1) and corresponding relaxivities (r_1) were evaluated after storage under identical conditions.

As summarized in Table S1, only minimal variations in r_1 values were observed for both CMD–DOTA–Mn(II) and CH–DOTA–Mn(II) after seven days at 4 °C and RT:

- CMD–DOTA–Mn(II): slight increase in r_1 (11.75 → 12.36 $mM^{-1}\cdot s^{-1}$)
- CH–DOTA–Mn(II): essentially unchanged (12.14 → 12.11 $mM^{-1}\cdot s^{-1}$)

These findings indicate that the coordination environment of Mn(II) remains intact, and no significant demetallation or structural degradation occurs. The relaxometric performance is preserved over time.

Table 1. Longitudinal relaxivities (r_1) and average hydrodynamic diameter of CMD-DOTA-Mn(II) and CH-DOTA-Mn(II) conjugates. DLS measurements were carried out after incubation for seven consequent days at room temperature (RT). In vitro relaxometric studies at 4°C and at RT were conducted for the same period of time.

**Conjugate**	**r_1 ($mM^{-1}\cdot s^{-1}$)** At 4° C in darkness	at RT	**Hydrodynamic Size (nm)*** At Day 0	at Day 7
**CMD-DOTA-Mn(II)**	**11.75**	**12.36**	**6.70± 0.013**	**7.02± 0.019**
**CH-DOTA-Mn(II)**	**12.14**	**12.11**	**4.79± 0.050**	**5.30± 0.840**

*DLS data represent average values from three independent measurements. Experimental conditions: viscosity = 0.8872 cP; refractive index (RI) = 1.330.

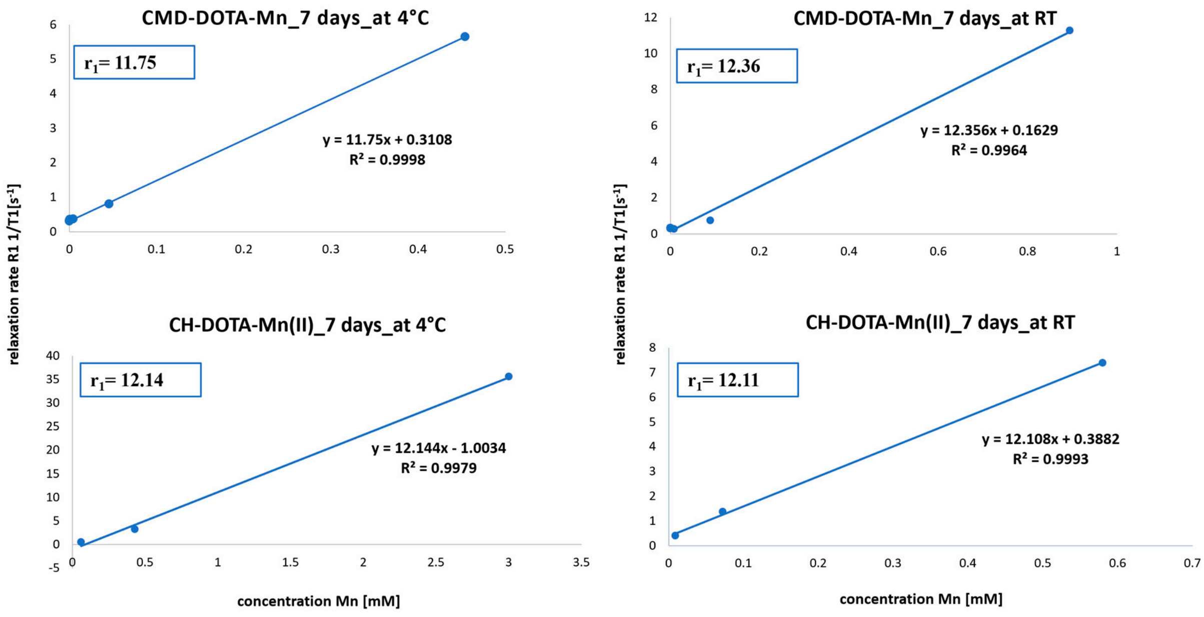

Figure S3. Plots of relaxation rates (R_1, [s^{-1}]) as a function of Mn concentration [mM] for CMD-DOTA-Mn(II) (first row) and CH-DOTA-Mn(II) (second row) after 7 days storage at 4° C in dark and at room temperature (RT), no exclusion of light. R_1 values were calculated from T_1 measurements obtained by ROI-based analysis of phantom images, where mean T_1 values and corresponding standard deviations (SD) were derived from signal distributions within each ROI. Error propagation from T_1 to R_1 ($R_1 = 1/T_1$) was

performed using Gaussian error propagation. The resulting uncertainties in R_1 were <1% and are therefore not visually distinguishable within the scale of the plot.

Overall, the combined DLS, visual inspection, and relaxometric data demonstrate that CMD–DOTA–Mn(II) and CH–DOTA–Mn(II) exhibit excellent short-term stability in physiological saline over seven days. The absence of aggregation, minimal size variation, and preserved relaxivity collectively support the structural integrity and kinetic stability of the Mn(II) complexes.

These findings provide strong experimental evidence that the investigated macromolecular systems maintain their physicochemical and functional properties under conditions relevant for preclinical and potential in vivo applications.

Disclaimer/Publisher's Note: The statements, opinions and data contained in all publications are solely those of the individual author(s) and contributor(s) and not of MDPI and/or the editor(s). MDPI and/or the editor(s) disclaim responsibility for any injury to people or property resulting from any ideas, methods, instructions or products referred to in the content.

https://doi.org/10.3390/pharmaceutics18050530
